# Meanings of quality of life held by patients with colorectal cancer in the context of chemotherapy^1^

**DOI:** 10.1590/0104-1169.0455.2572

**Published:** 2015-07-03

**Authors:** Luciana Scatralhe Buetto, Marcia Maria Fontão Zago

**Affiliations:** 2 PhD; 3 PhD, Associate Professor, Escola de Enfermagem de Ribeirão Preto, Universidade de São Paulo, PAHO/WHO Collaborating Centre for Nursing Research Development, Ribeirão Preto, SP, Brazil

**Keywords:** Quality of Life Related to Health, Chemotherapy, Colorectal Neoplasms, Anthropology Medical, Anthropology, Cultural, Oncological Nursing

## Abstract

**OBJECTIVE:**

this study's aim was to interpret the meanings assigned to quality of life by patients with colorectal cancer undergoing chemotherapy.

**METHOD:**

the ethnographic method and the medical anthropology theoretical framework were used. Data were collected through semi-structured interviews and participant observations with 16 men and women aged from 43 to 75 years old undergoing chemotherapy in a university hospital.

**RESULTS:**

the meanings and senses describe biographical ruptures, loss of normality of life, personal and social suffering, and the need to respond to chemotherapy's side effects; chemotherapy is seen as a transitional stage for a cure. Quality of life is considered unsatisfactory because the treatment imposes personal and social limitations and QoL is linked to resuming normal life.

**CONCLUSIONS:**

the meanings show the importance of considering sociocultural aspects in the conceptualization and assessment of quality of life.

## Introduction

Cancer populates the social imaginary with representations of suffering, helplessness, loss and finitude. Even with increased survival rates for cancer patients, there are still challenges to demystifying beliefs about the disease and its treatment^1^.

Cancer is the second main cause of morbidity and mortality for the Brazilian and world populations, thus constitutes a public health problem. Estimates of the José Alencar Gomes da Silva National Institute of Cancer (INCA) indicate there will be 576,000 new cases of cancer in Brazil for 2014/2015. Of these, 33,000 will be colorectal cancer (CRC), which equally affects both sexes among individuals over 50 years of age^2^.

CRC etiological factors include heredity, aging, diet, and prior inflammatory colorectal diseases^3^.

In most cases, surgery is the first stage of treatment and results in changes in the patient's body image and social activities due to a temporary or permanent stoma^4^.

Chemotherapy is the second therapeutic option. It consists of the use of anti-neoplastic drugs with systemic action applied through various routes of administration to destroy tumor cells or alleviate symptoms. In the case of CRC, chemotherapy is a neo-adjuvant, adjuvant and/or palliative treatment^5^.

Monoclonal antibodies with the ability to recognize and bind to specific tumor antigens are also employed, triggering immunological responses capable of sparing normal cells and reducing the adverse effects arising from the toxicity of conventional anti-neoplastic drugs^6^.

Chemotherapy is also relevant in the treatment of CRC, creating an expectation and a hope for a cure, but the belief still persists that this is the worst of the anti-neoplastic treatments due to its adverse reactions^6^^-^ 7.

Health-related quality of life, or simply quality of life (QoL), in the presence of chronic conditions such as CRC is a growing concern among health workers who seek to orient decision-making concerning care interventions^8^. The World Health Organization defines QoL as the perception of individuals concerning their position in life in the context of values with which they live and in relation to their objectives, expectations and concerns. This concept comprises aspects concerning physical and psychological health, level of functionality, sociability, and relationships with environmental characteristics. There is agreement among workers that this concept is subjective, multidimensional and includes elements of both positive and/or negative assessments^9^.z

In recent years, studies^10^^-^ 11 assessed the QoL of CRC patients using different measures such as the Functional Assessment of Cancer Therapy-Colorectal (FACT-C). Some studies report satisfactory QoL, while others report unsatisfactory QoL. The authors of one integrative review^10^ observed an association between the QoL of individuals with CRC with physical exercise, follow-up with exams, psychosocial assessment, and aspects inherent to people. A cancer diagnosis and its treatment affect various domains, but studies do not report strong evidence. The authors of a systematic review^12^ that was concluded five years after surgery, reports that individuals experienced poor physical QoL, obtained worse scores for depression, and reported intestinal problems and anxiety due to the possibility of recurrence.

We share a criticism^13^ that when applying instruments to measure QoL, one dissociates the consequences of the disease and treatment, the implications of economic aspects, social and family life, social roles and prospects for the future. Hence, one needs to understand and interpret the sociocultural dimension of QoL^14^.

Nurses face challenges when providing care to patients with CRC, which involves technical and humanist knowledge. It is essential that workers be aware of how cancer patients experience their disease, treatment and the various sociocultural processes involved, such as QoL. The anthropological perspective can contribute to this understanding and broaden knowledge in the application of integral nursing care.

This study is guided by the following questions: What are the meanings assigned to QoL during chemotherapy treatment of CRC? How do patients perceive their QoL to be affected? Why do they think like this? The objective is to interpret the meanings assigned to QoL by patients with CRC undergoing chemotherapy.

## Method

The study was developed according to the ethnographic methodology and medical anthropology.

Medical anthropology follows an interpretative approach, the central concept of which is culture as a pattern of meanings incorporated into symbols, and, through these symbols, people communicate, develop knowledge and their actions in social life. This theoretical approach proposes understanding and interpreting human meanings, while valuing the subjectivity of the disease for the subjects, treatments and QoL. From the perspective of culture as a symbolic system, health and disease are a continuum of events, and people understand these events through this system^15^.

Ethnography deals with scientific descriptions aiming to interpret the meanings of reports and actions of CRC patients undergoing chemotherapy by integrating them with the cultural context^14^^-^ 15.

The project was approved by the Institutional Review Board (Protocol No. 1412/2011) and all the patients signed free and informed consent forms. All names are fictitious to ensure confidentiality.

The participants were contacted in the coloprotology outpatient clinic of a university hospital in the interior of the state of São Paulo, Brazil. Data were collected on the service's premises and at the patients' homes, from March 2012 to July 2013.

The techniques of data collection included participant observation and video-recorded, semi-structured interviews guided by the questions: What is your life like with the treatment? At this point, what is QoL for you? What influences your QoL during your chemotherapy? These questions were broadened to deepen the patients' reports. A total of 36 interviews and observations were conducted; each lasted 70 minutes on average.

The transcriptions of the reports, observations and field diary notes were integrated into texts and submitted to inductive thematic analysis^16^. Data are coded in this process without fitting it into preexistent categories. The analysis is oriented to the identification of themes and patterns of latent or implicit meanings and senses that go beyond the bare content.

The ethnographic analysis of data followed two main analytical processes: the identification of meanings and the construction of the thematic core. The meanings are the descriptions of the process experienced by the patients in terms of their ideas and actions concerning the disease, treatment, especially chemotherapy, and QoL, explained through common and/or significant motivations and justifications extracted from their cultural histories. Based on the meanings, we constructed the core of meanings through themes based on the theoretical framework. The hermeneutic circle was applied when the researchers interacted with data collected from each participant and the whole data set, identifying themes and relating them with theory and the literature to prepare grounds for the meanings expressed and relativized for the context under study^14^.

To ensure the study's accuracy, engagement with the study participants was extended. Individuals were included in the study regardless of sex, age, socioeconomic or educational status in order to obtain different perspectives. Many observations and interviews were performed using the same questions at different points in time. The participants' social and clinical characteristics were considered in the analysis and interpretation of data and discussed among the researchers^17^.

The participants included four men and 12 women, aged from 43 to 75 years old, living in the city where the study occurred and the surrounding region, with educational levels ranging from incomplete primary school (6) to a bachelor's degree (3). Their occupations varied, but most were of low social status: carpenter, hairdresser, pamphleteer, domestic worker, and homemaker, among others. All participants had been diagnosed with tubular adenocarcinoma for at least six months; four patients were in stage II, three in stage III, and nine in stage IV; nine had hepatic and/or lung metastases. Ten were undergoing chemotherapy with an oxaliplatin and/or capecitabine regimen; three were only using capecitabine; one patient was taking 5-Fluoracil and folinic acid; one was receiving a combination of oxaliplatin, 5-Fluoracil and bevacizumabe; and one patient was receiving a combination of oxaliplatin, capecitabine and bevacizumabe.

## Results and discussion

### The meanings assigned to the disease, chemotherapy and QoL

One of the meanings most frequently stressed by patients was the moral aspect of CRC given their past lives. *If I hadn't drunk and smoked so much, this wouldn't have happened *(Mário, 59 years old); other times, it was seen as a consequence of a family problem that was not solved. *My cancer is a result of hurt feelings! I had to give up my life and take care of my family. As they say, grief turns into disease* (Tatiane, 47 years old). From a layman's perspective, the explanations of the cause of the disease may involve psychological, social, spiritual and/or biological aspects assigned to moral behavior and an overload of emotions^1^.

After the diagnosis of a socially stigmatized disease, the patients prepare to face treatment. The first step is surgery, a procedure initially considered to be sufficient to eradicate CRC. *When I went into surgery, I thought I had a tumor, not cancer! I thought that the disease would end there with the surgery *(Taciana, 53 years old). From the news the patient needed a stoma, whether a temporary or permanent one, emerged a conflict between living and dying. *I knew there would be no way. I had to have the surgery and use the bag, otherwise I'd die. It was my chance to stay alive* (Daniela, 45 years old). Resignation^4^, a passive process to deal with the chaos of the situation, is the way found by these participants to deal with their body image and altered bowel function.

The need for chemotherapy emerged as the disease progressed to ensure the surgery's success and a new challenge was imposed. *The news that I would have to undergo chemotherapy was worse than the news I had cancer! *(Cristiane, 53 years old); *I think the tumor grew again and I have to undergo the chemotherapy to kill its roots *(Tatiane, 47 years old). These reports show a belief in the suffering caused by the therapy that either heals or kills, based on a commonplace belief.

The physiological reactions to cancer drugs are what scare patients the most. *I knew it wasn't simple. You can't imagine all that! *(João, 49 years old); *The first ten days were terrifying. I thought that my body would get used to the effects but no, it didn't!* (Daniela, 45 years old).

After initiating chemotherapy, the participants expected its adverse events, which were known to be devastating, such as alopecia, nausea, vomiting, which are common to the various medication schemes used. *I vomited, got dizzy all day *(Daniela, 45 years old)*; I thought that my hair would fall out, that I'd go bald *(Mário, 59 years old). The nausea and vomiting imposed changes in diet. *I got to a point when I'd only have rice broth, which was the only thing that would stay in my stomach. I had to make many changes in my diet. *(Cristina, 72 years old).

The side effects experienced by those receiving chemotherapy with monoclonal antibodies are different, but also disabling. *The physician told me about the side effects, the skin of the hands and feet gets rough and dark. I got many canker sores and my feet got really bad (*Mario, 59 years old)*; My feet's skin got pretty thin and started shedding, then they cracked a little but my hair didn't fall out! I got lucky! *(Rogério, 48 years old).

The duration of the chemotherapy is a concern for patients. *I can't wait for all this to be over! *(Cristiane, 53 years old). Side effects worsen the suffering, even when they are temporary. Patients endure the adverse reactions for they are less severe than the disease.

Fatigue is another side effect all the participants reported. *I can't do anything as I did before. Now, everything is slower* (Cristina, 72 years old); *I feel pretty weak. It's difficult even to get up from the bed *(Maria Helena, 75 years old).

The patients' rational natures leads them to associate physical resistance with their bodies' resilience as if it were a type of bargain with God, themselves and society; this manner of thinking and acting maintains hope in the treatment^18^. *There is no point in complaining; you have to accept it, undergo the treatment. Everything will be all right*(Helena, 60 years old);* I have to stay positive so I can finish the treatment soon* (Cristiane, 53 years old).

Even with limitations, the patients feel a moral obligation to overcome the process. *I can deal with it. I'm having heartburn today, but it's not too bad *(Helena, 60 years old). This practice of overcoming limitations and minimizing the disease and chemotherapy's adverse events is considering themselves not to be sick and taking into account the possibility of a cure. *I don't think about the disease. I don't know whether I haven't realized it yet. I want to believe I'll be cured* (Taciana, 53 years old); *I don't feel sick; let's wait for the chemotherapy to be over* (Maria, 57 years old). This coping strategy follows a logic built upon personal experiences with other patients, family members, and the social group, to deal with what does not have an explanation from a commonplace belief point of view, reinforcing one's individual identity and place in the social body^18^.

Nevertheless, difficulty in keeping up with work or even home chores is what the patients most frequently lamented, regardless of sex or age. *I'm a hairdresser, had a saloon. My focus was always my work. It's difficult for me to know that I may have to close my saloon. I can't live on retirement pay alone. My life has changed a lot!* (Lucimara, 43 years old); *I'm still involved with the workshop; I take care of the clients' budgets. When my clients negotiate a big project, they want to talk to me and I have to show I'm OK! *(João, 49 years old). It is difficult for patients to imagine that their family members or other people consider them less capable to respond to their personal and social responsibilities, to take care of the family's financial support; it is difficult being labeled as a cancer patient and having people feeling sorry for them. A sociocultural practice used in this situation is social isolation, restricting activities to the family space, because this somewhat alleviates their suffering^4^^,^ 18.

The relationships of couples are also affected, but only one patient addressed the issue. *We don't have sex anymore! He won't touch me, I guess it's out of fear. He says he's afraid of hurting me* (Helena, 60 years old).

All the participants attributed the possibility of a cure as a meaning of chemotherapy*. I don't blame chemotherapy for the changes that took place in my life, I blame the disease. Chemotherapy means a cure! Always a chance of a cure!* (Helena, 60 years old).

The therapy was not successful for nine of the participants and the confirmation that the disease had reached metastases was a landmark. *Six months ago I underwent surgery. I'm doing the chemotherapy but new tumors have already appeared*(Lucimara, 43 years old); *I don't know about the future! *(Cristiane, 53 years old). For these individuals, finitude is something they think about. *I don't want to die! *(Maria, 57 years old). These reports corroborate the results of one American study that determined 81% of its participants undergoing chemotherapy to treat stage IV CRC did not understand the treatment was only palliative, even though the medical staff had informed them of the nature of the treatment. The authors also clarified that there was no association among educational level, functional status and the decision to undergo chemotherapy with unrealistic beliefs concerning the therapy^19^, as observed in this study. Nevertheless, the patients are hopeful they will resume normal life. *I want to go back to my activities, socialize. I want my life back!* (Cristina, 72 years old).

These meanings show that surgery and chemotherapy generate ruptures in the lives of patients^4^, along with personal and social distress. The prescription of chemotherapy is an attempt to allow patients to resume normal life. Patients use the lay rationality, influenced by the biomedical model, with justifications and motivations that give meaning and support their practice to deal with what are experiencing. This rationality reveals the relationships among individual, social, cultural and spiritual worlds. The logic is to fight against disease and the treatment's adverse events, seeking strategies to avoid suffering^20^.

Patients expect to recover their individual bodies and social lives because they deem their bodies to be resilient and themselves able to maintain control over life. Despite the problems and disappointments with the treatments, these individuals are still able to expect that *something good will happen to me* (Helena, 60 years old). Hope gives them strength to keep fighting and to resume their life projects. Having hope is a cultural characteristic of humans and corresponds to the moral/social expectation of never giving up^21^.

From the perspectives of the participants regarding the experience of having CRC, chemotherapy is a transitional step to healing. This process defines a process from disease to cure; an undefined period that accompanies the patient to a socially expected situation - having cancer and being cured^18^.

These meanings highlight what is valued and what needs to be done in daily life to survive, preserve self-esteem and a sense of normality^22^, which is common to the results reported by other qualitative studies addressing this topic^23^^-^ 24.

The meaning of QoL is exemplified in the following reports. *I don't know how to explain what QoL is. I only think about getting better and not having the disease anymore, not needing to undergo chemotherapy anymore. QoL is being healthy, able to work, to live peacefully, that's it!* (Mario, 59 years old); *It's going back to what was normal for me! *(João, 49 years old). The attributes associated with QoL are related to having a normal life, not being sick, being independent and able to work. Labor, even domestic chores, implies a moral value among groups of patients from the urban Brazilian society^4^^,^ 14. This meaning is constructed in the relationships that exist among people, including personal judgments, regarding one's current life, experiences, and prospects for the future.

### The meanings of QoL

It was difficult for the participants to talk about QoL due to its generic use in everyday language. One of the themes most frequently mentioned by the patients was the importance of going back to a normal pattern of life, from the conceptual perspective of QoL. *I'm not sure whether I'll be normal again* (Maria 57 years old). For Brazilian chronic patients, normality means to be healthy as they were before the disease developed and being able to play their social roles, especially work, social relationships, within the family and in relation to leisure^4^. This category is an attribute of health, which emphasizes disease as a justification for losing control over current life.

In the beginning, chemotherapy did not impede everyday activities, but with time all activities became restricted, even home chores. In this context, there is a personal and social expectation. *Society requires me to be well because it influences the quality of the woodwork we provide. It is from this workshop that we take our sustenance and that of the families of my employees. The disease and treatment changed the lives of all of us* (João, 49 years old); *I have energy to do almost nothing. I don't feel like doing anything* (Alice, 62 years old).

Even feeling disappointed with unsuccessful chemotherapy, there is hope for a second chance, and thus they must keep fighting. The participants expect to recover their individual and their social bodies because they consider their bodies to be resistant.

These meanings assigned to the disease and chemotherapy seem to be a moral experience^22^. By moral, we note what is valued or what needs to be performed in everyday life to survive, in the right/wrong, good/unfair continuums that are advocated in the social world. In addition to personal and social suffering, the moral aspect of being unproductive generates uncertainty and self-preservation in patients.

In this context, the body is not only a means to relate with the world, it is a means of identification among those with CRC undergoing chemotherapy and can be an obstacle for the maintenance of their identities and social integration. To deal with culturally unaccepted limitations, patients use a compensatory logic, trying to resign themselves to the moral experience enveloping them, considering the normality of their past but hoping they will be cured in the future. In this compensatory rationale^14^, social reintegration, which would ensure QoL, is related to* going back to what was normal*, *being able to work, being whole*, as a process integrated into the participants' experiences of becoming sick, regardless of their social or educational characteristics, gender or age, because life is the maximum symbolic value of humankind^7^^,^ 14.

These meanings show that that participants experience unsatisfactory QoL due to the loss of control of life caused by the disease and by chemotherapy's side effects. They perceive that this therapy does not ensure a cure and that, at the end of the treatment, they remain uncertain of the future, despite all the suffering they morally experienced. The conceptual elements of QoL, from the perspective of patients, show the inter-relationships of behaviors and human values with the social and disease contexts^8^^,^ 13 ^,^ 21 ^,^ 25.

These results highlight that QoL is a subjective concept dependent on various dimensions of life; it is dynamic, because it changes over time due to the progression of the disease, inclusion of new therapies, the patient's internal ability, changes of values and actions, and is essentially an individual judgment^8^^,^ 13 ^,^ 24.

In this manner, the use of theoretical-methodological interpretative approaches, which focus on the sociocultural context, the situations experienced in the healthcare system, as well as the therapeutic actions and consequences, favor deepening the analysis and discussion of conceptual elements of QoL, during cancer treatment.

It is important to reflect on how to concretize the quality and quantity of conceptual elements of QoL. Quality cannot take the place of quantity, nor the reverse. Even though quantity is a type of quality, the contrary is not true. Instruments of QoL, in general, measure quantity and not quality^8^.

Considering the prevalence of CRC in Brazil, a lack of clinical response with the use of current therapies given high rates of unsatisfactory outcomes means other indicators are needed, in addition to QoL, to support the maintenance or prescription of treatments in different stages of the disease.

On the other hand, when considering the analysis of QoL among people with cancer, one needs to pay attention to the ethical aspects of results, because this assessment involves the lives of other people^8^.

The anthropological perspective ensured consistency of data and the respective analysis through fieldwork, participant observation, and in-depth interviews. These facilitated the proximity of researchers to situations experienced by the participants, as well as the construction of meanings of QoL^8^.

## Conclusion

A group of people with CRC undergoing chemotherapy was addressed in this study to understand the meanings and senses assigned to QoL during treatment. The ethnographic method and interpretative medical anthropology were used.

The identification of meanings assigned to cancer and therapy enabled experiences to be described in a way that includes: a new identity of an individual with cancer and a response to the diagnosis; the participants' beliefs concerning the disease; the changes in the body caused by surgery; the biographical ruptures the patient experiences; the valorization of normality of life experienced prior to the disease; suffering and the need to respond.

The patients recognized that surgery was not effective and that chemotherapy would be necessary. This therapy generated new ruptures and limitations, with repercussions in the personal, social and familial spheres. This experience was revealed to be a period of intense suffering, with the meaning of struggle for life. The participants sought ways to adjust to their new situations, despite uncertainty about the future.

The meanings concerning cancer and its treatments implied a moral experience due to the personal and social suffering caused, so patients tried to preserve a sense of self-esteem, rejecting the impossibility of cure. In this context, chemotherapy is a transitional process that transforms life, leading to losses, but having value in maintaining hope in cure, despite adverse events related to it.

From these meanings emerged the main thematic core: quality of life as a possibility to resume normal life as it was prior to the disease. QoL is presented in relation to a new personal and social identity, marked by a loss of control over life, by a limitation of the ability to work, which is highly valued by these people as a possible means to preserve their autonomy. Its meaning is related to resuming one's personal and social life as it was prior to the disease's onset.

From the perspective of patients, QoL during chemotherapy, regardless of sex, occupation or age, is expressed as a critical symbolism of the disease due to the moral experience and not due to its treatment. It is related to the patients' ability to manage social roles, especially to resume work and to feel normal, which in the context under study, was considered unsatisfactory.

Given the previous discussion, we suggest greater investment in the use of scientific knowledge, to acquire different and complementary perspectives, that is, to develop qualitative studies addressing the experience of QoL with cancer to combine different dimensions to quantitative results.

The meanings presented show the importance of broadening the view of QoL on the part of nurses providing care to patients with CRC, including sociocultural references from the context of people living the disease and chemotherapy, going beyond the biological and technical dimensions of care delivery.
